# Depletion of nerve growth factor in chemotherapy-induced peripheral neuropathy associated with hematologic malignancies

**DOI:** 10.1371/journal.pone.0183491

**Published:** 2017-08-21

**Authors:** Jeonghwan Youk, Young-Sook Kim, Jung-Ah Lim, Dong-Yeop Shin, Youngil Koh, Soon-Tae Lee, Inho Kim

**Affiliations:** 1 Division of Hematology/Oncology, Department of Internal Medicine, Seoul National University Hospital, Seoul, South Korea; 2 Department of Neurology, Seoul National University Hospital, Seoul, South Korea; 3 Cancer Research Institute, Seoul National University College of Medicine, Seoul, South Korea; Weill Cornell Medical College in Qatar, QATAR

## Abstract

**Objective:**

To investigate whether the depletion of nerve growth factor (NGF) is associated with the development of chemotherapy-induced peripheral neuropathy (CIPN) in patients with hematologic malignancy.

**Methods:**

We prospectively enrolled hematologic cancer patients who had a plan to receive bortezomib, thalidomide, or vincristine. Baseline NGF levels were measured within one week before the start date of chemotherapy. Follow-up NGF levels were measured after four months from the start date of chemotherapy or the date when CIPN was initially diagnosed.

**Results:**

Baseline and follow-up NGF pairs were measured in 45 patients (male/female = 27/18, median age = 63 years old). CIPN has developed in 28 patients. In the CIPN group, the level of NGF was significantly decreased after chemotherapy compared to the baseline (△NGF = −3.52 ±5.72; *p*-value = 0.003), while the NGF level of the no-CIPN group was not changed after chemotherapy. The differences in △NGF levels between the CIPN and no-CIPN group were more profound when analyzed in the subgroup of newly diagnosed multiple myeloma patients (△NGF = −4.14 ± 4.87 pg/ml for the CIPN group and +2.52 ± 8.39 pg/ml for the no-CIPN group; *p*-value = 0.043).

**Conclusions:**

This study shows that the depletion of NGF occurs during the development of CIPN, suggesting pathogenesis based on the role of NGF and therapeutic implications.

## Introduction

Chemotherapy-induced peripheral neuropathy (CIPN) is one of the major complications of chemotherapy in hematologic malignancy, which deteriorates quality of life (QOL)[1]. It frequently causes distal sensory neuropathic pain, chronic general weakness and early discontinuation of chemotherapeutic agents, which can increase cancer-related morbidity and mortality[2, 3]. Some mechanisms of CIPN have been investigated. It has been found that taxane and vincristine perturb axonal cytoplasmic flow in affected neuronal cells, and bortezomib inhibits NF-kB activation, which in turn is likely to block the transcription of neurotrophins to regulate neuronal survival[4–8].

One of major neurotrophins is nerve growth factor (NGF), known to regulate the growth, maintenance, and survival of neurons[9, 10]. This factor is trophic to small fiber neurons that dominate pain, temperature, and autonomic function[11]. The correlation between a reduced NGF level and the occurrence of diabetic neuropathy has been well documented[11]. The role of NGF has also been investigated in patients with CIPN. Patients with CIPN showed reduced serum NGF levels in neuropathic cancer patients[12, 13]. In animal models, exogenous NGF administration showed the possibility of preventing cisplatin and paclitaxel-induced peripheral neuropathy[14–18]. However, most previous CIPN studies related to NGF were limited to conventional cytotoxic agents.

In the last two decades, bortezomib and thalidomide have been widely used in hematologic malignancies. More than 90% of patients who were treated with combination of the two drugs have suffered from CIPN[19]. However, the relationship between NGF level and CIPN in patients with hematologic malignancies remains elusive. Thus, we prospectively designed this study to identify whether the depletion of NGF occurred during the development of CIPN in hematologic cancer patients.

## Materials and methods

### Patients and samples

Patients who had a plan to receive a new line of chemotherapy, including bortezomib, thalidomide, and vincristine, and had not suffered from CIPN were prospectively enrolled from August 2014 to February 2016. The patients were diagnosed with hematologic malignancies between February 2010 and January 2016 at Seoul National University Hospital. Baseline NGF levels were measured within one week before the first cycle of chemotherapy. Follow-up NGF levels were planned at the fourth month after the initiation of the chemotherapy or when the patients felt symptoms of CIPN, including neuropathic pain or numbness. The clinical diagnosis of CIPN was made by an experienced neurologist (S.-T.L.). To exclude other peripheral neuropathies, we performed laboratory screening tests including complete blood cell count, comprehensive metabolic profile, hemoglobin A1c, vitamin B12 level, erythrocyte sedimentation rate, C-reactive protein, human immunodeficiency virus, and hepatitis virus. All subjects were provided with written informed consent. This study was approved by an institutional review board at Seoul National University Hospital (H-1407-182-598).

### Measurement of serum NGF

To measure NGF levels, 2 milliliters of blood was drawn from peripheral veins of patients. Both baseline and follow-up blood samples were immediately transported to the central laboratory in SST^TM^ serum separation tubes (Becton, Dickinson and Company, Franklin Lakes, NJ, USA). Blood samples were left at room temperature for 30 minutes to allow complete coagulation. Coagulated samples were then centrifuged at 1,300 g (2500 rpm) for 10 minutes at 4°C to separate serum. Serum was transferred to a new CryoTube (Sigma-Aldrich, St. Louis, MO, USA). Serum samples were immediately frozen at −80°C and stored until analysis. NGF concentrations in serum samples were measured using an enzyme-linked immunosorbent assay kit (ChemiKine Nerve Growth Factor Sandwich ELISA Kit #CYT304, Merk Millipore, Darmstadt, Germany) following the manufacturer’s instructions. The measurement was performed in duplicate.

### Response evaluation

We evaluated the response of chemotherapy according to the types of disease. For multiple myeloma, the International Myeloma Working Group criteria were used [20]. For lymphoma, the Lugano classification was applied [21]. In the leukemia cases, five percent or more of blast counts in follow-up bone marrow examinations were considered persistence, and less than five percent of blast counts was considered complete remission.

### Subjective pain score evaluation

To evaluate correlation between NGF levels and subjective pain scores, patients complaining of CIPN were asked to fill out the Brief Pain Inventory-Short Form (BPI-SF) questionnaire at the time when neuropathy was developed [22]. The BPI-SF consisted of nine questions, including average pain score and the worst pain score over the last 24 hours.

### Statistical analysis

All categorical data were analyzed using Pearson’s Chi-square test or Fisher’s exact test. Normal distribution was determined by Shapiro-Wilk test. For continuous variables, Student’s t-test, paired-t test, Mann-Whitney U, or Pearson’s correlation test were used. In subgroup analysis, non-parametric analysis was used for the component in which the number of patients was less than ten. To compare the variance of two data sets, an F test was performed. All statistical calculations were performed using R version 3.1.3 (R Core Team, Vienna, Austria). A P-value of < 0.05 (two-tailed) was considered statistically significant.

## Results

### Characteristics of the patients

A total of 73 patients (male/female = 44/29, median age = 62 years old) were enrolled, and baseline NGF levels were measured. We were able to check follow-up NGF levels in 45 patients (male/female = 27/18, median age = 63 years old, Table 1) because the other 28 patients were lost to follow-up. Among the 45 patients, 32 (72%), 11 (24%), and 2 (4%) were diagnosed with multiple myeloma, lymphoma, and acute leukemia, respectively. Before this study, 18 (40%) of the patients received previous chemotherapy, including bortezomib, thalidomide, vincristine, or autologous stem cell transplantation (ASCT).

**Table 1:** Baseline clinical and laboratory characteristics of the patients.

	Total (n = 45)	No-CIPN (n = 17)	CIPN (n = 28)	*p*-value
Median age, years [range]	63 [42–82]	60 [42–82]	65 [46–80]	0.261
Gender (M/F)	27/18	11/6	16/12	
Baseline M-protein, median [IQR] (g/dl)	1.81 [0.48–4.66]	1.28 [0.06–3.06]	2.54 [0.77–4.68]	0.253
Follow-up M-protein, median [IQR] (g/dl)	0.37 [0.00–0.79]	0.26 [0.00–0.53]	0.41 [0.00–0.84]	0.511
Previous ASCT	17	10	7	0.051
Underlying diabetes mellitus	4	2	2	0.626
β-2 microglobulin, median [IQR], μg/ml	3.57 [2.60–7.96]	3.81 [2.52–9.46]	3.57 [2.69–7.59]	0.879
Albumin, mean (SD) g/dl	4.00 [3.60–4.30]	3.95 (0.54)	3.91 (0.53)	0.879
Disease				1
Multiple myeloma	32	12	20	
Lymphoma	11	4	7	
Acute leukemia	2	1	1	
Subtypes of multiple myeloma				
Heavy chain				0.274
IgG	18	8	10	
IgA	5	3	2	
Light chain disease	9	1	8	
Light chain				0.504
Kappa	17	8	9	
Lambda	15	4	11	
Previous treatment				1
No treatment	27	11	16	
Velcade only	6	2	4	
Thalidomide only	6	2	4	
Vincristine only	5	2	3	
Combination	1	0	1	
Intervention during this study				0.694
Velcade only	22	7	15	
Thalidomide only	7	2	5	
Vincristine only	10	5	5	
Combination	6	3	3	
Response				0.767
CR	10	3	7	
PR	26	11	15	
Others†	9	3	6	

ASCT = autologous stem cell transplantation. CR = complete remission, and PR = partial remission.

†Others include stable disease, progressive disease, and persistence.

In total, 28 (62%) of the patients had newly developed CIPN during this study. Except for one patient, CIPN occurred within 4 months after the initiation of neuropathic chemotherapy. When patients were grouped into whether CIPN occurred (the CIPN group, n = 28) or not (the no-CIPN group, n = 17), the baseline and laboratory characteristics were well balanced in the two groups (Table 1). Among the patients who developed CIPN, the mean of the average neuropathic pain scores was 3.00 ± 1.44. The mean of the highest neuropathic pain score over the past 24 hours was 4.14 ± 2.40.

### NGF level changes in the CIPN and no-CIPN groups

**Fig 1:**
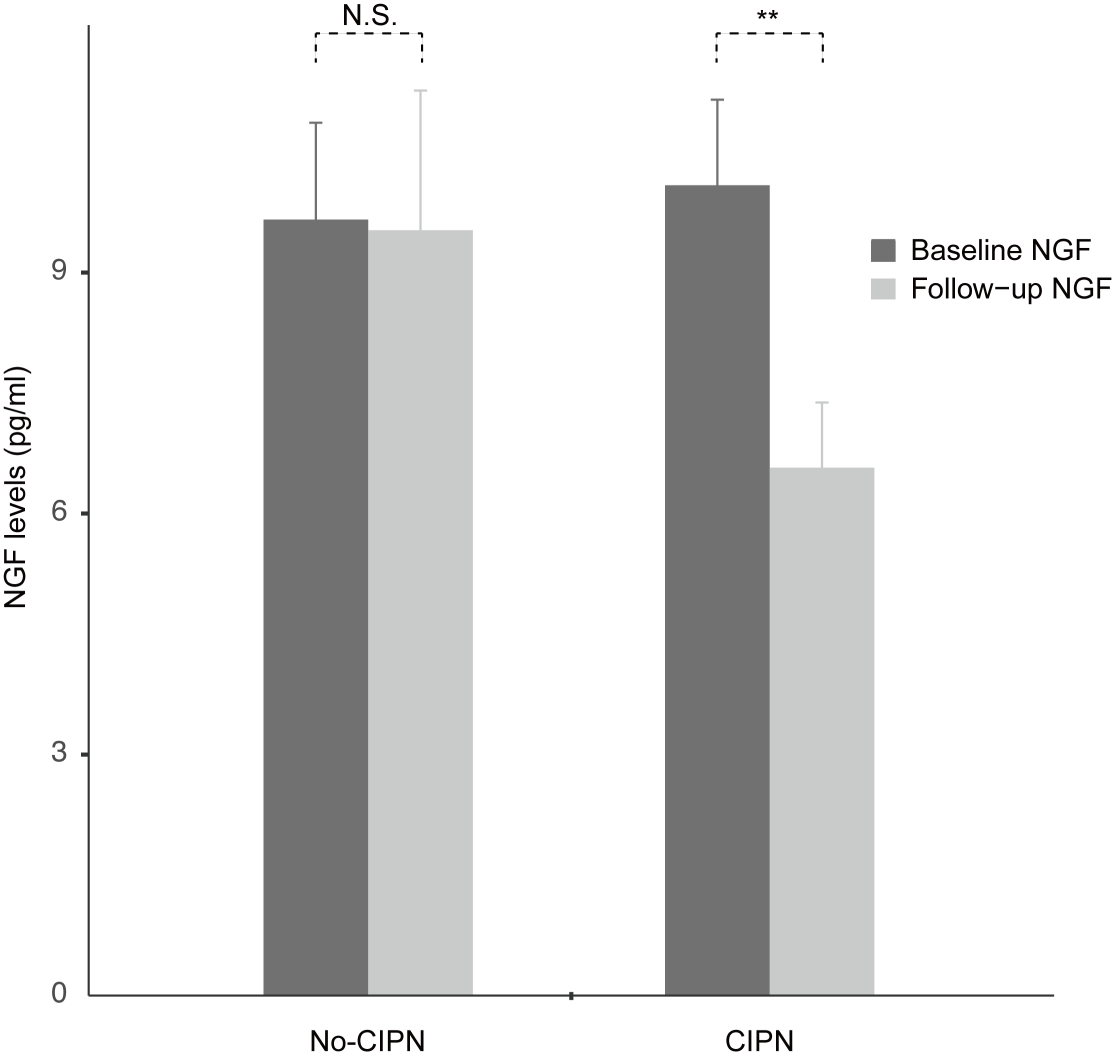
Bar-plot of the changes in NGF before and after chemotherapy. In the CIPN group, the NGF levels were significantly decreased after the chemotherapy. In the no-CIPN group, the changes in NGF were not significant. NS = not significant. ** P<0.01.

In the 45 patients, the baseline NGF and follow-up NGF levels were 9.92 ± 5.34 pg/ml and 7.69 ± 5.67 pg/ml, respectively. Using the paired t-test, NGF levels were significantly decreased after chemotherapy [△NGF (follow-up NGF level–baseline NGF level) = −2.24 ± 6.43; *p*-value = 0.024]. This result suggests that the chemotherapy decreases the NGF levels overall. Next, we compared the NGF levels between those with newly developed CIPN (n = 28) and no CIPN (n = 17). In the CIPN group, the baseline and follow-up NGF levels were 10.09 ± 5.64 pg/ml and 6.57 ± 4.30 pg/ml, respectively, and the difference was significant (Fig 1; △NGF = −3.52 ± 5.72; *p*-value = 0.003). In the no-CIPN group, the baseline NGF and follow-up NGF levels were 9.66 ± 4.98 pg/ml and 9.53 ± 7.17 pg/ml, respectively, and the difference was not significant (△NGF = −0.13 ± 7.13; *p*-value = 0.940). The baseline NGF levels between the two groups were not significantly different (*p*-value = 0.792), nor were the sampling time intervals (from baseline to follow-up; median days = 89 days for No-CIPN and 97 days for CIPN; *p*-value = 0.088). When we directly compared the changes of the NGF levels (△NGF levels) between the two groups using Student’s t-test, the △NGF levels in the CIPN group (△NGF = −3.52 ± 5.72) tended to be lower than those in the no-CIPN group (△NGF = −0.13 ± 7.13) but failed to show statistical significance (*p*-value = 0.087).

### Subgroup analysis

**Fig 2:**
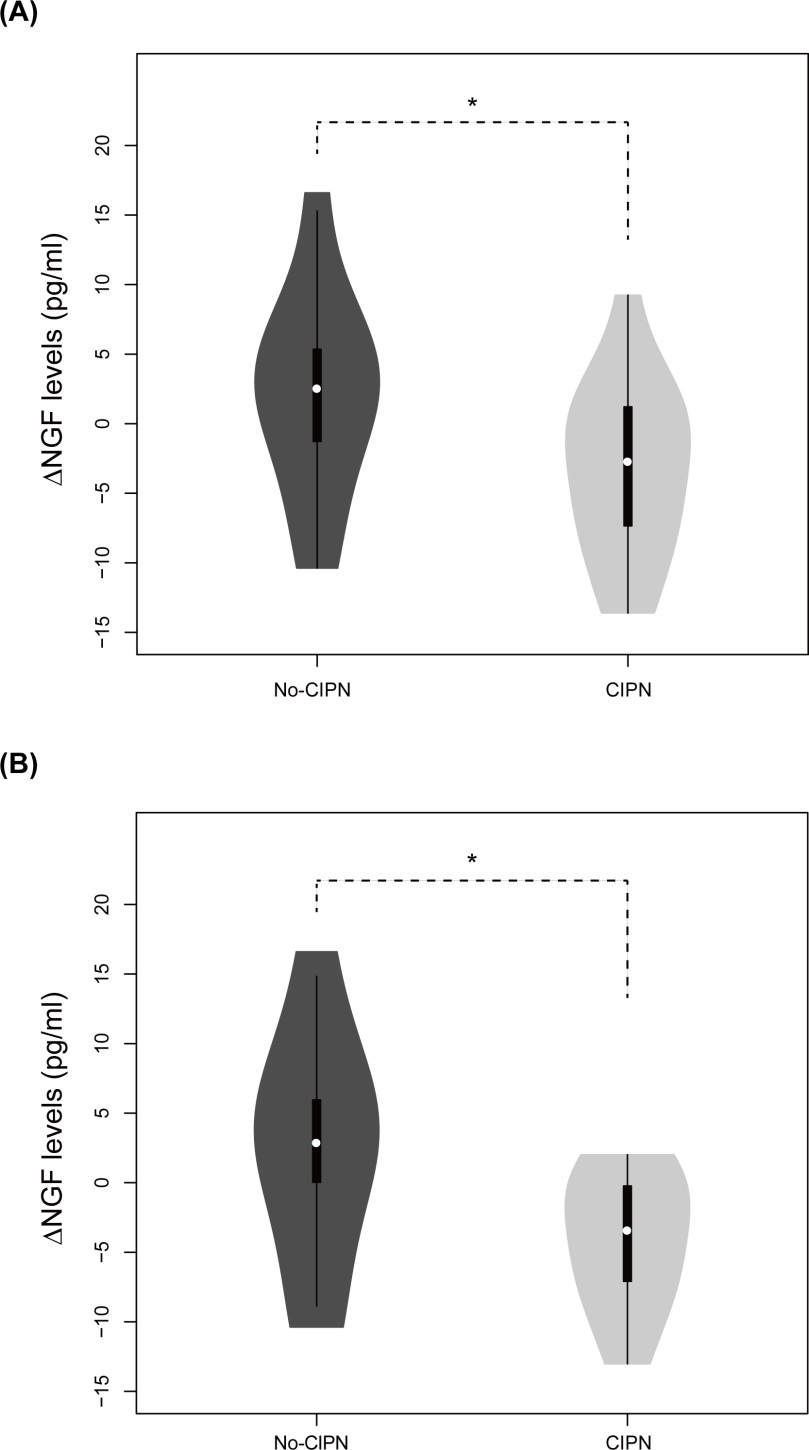
Violin-plot of NGF differences between baseline and follow-up measurements in patients without previous treatments. White circle markings indicate median level of △NGF, and thick black bars show interquartile range[23]. The NGF differences were significantly different between the two groups in (A) and (B) (*p*-value = 0.0476 and 0.0432, respectively). (A) Newly diagnosed patients were all involved. The number of patients was 11 and 16 in the no-CIPN and the CIPN groups, respectively. (B) Newly diagnosed multiple myeloma patients were involved. The number of patients was 8 and 11 in the no-CIPN and the CIPN groups, respectively.

Considering heterogeneous patients’ characteristics, subgroup analysis was performed to investigate factors influencing the NGF levels (Table 2). In the patients with previous chemotherapies, the △NGF was not significantly different between the two groups (n = 6 for no-CIPN and 12 for CIPN; *p*-value = 0.964). However, in patients without previous chemotherapy, the △NGF levels were significantly lower in the CIPN group compared to the no-CIPN group (Fig 2A; n = 11 for no-CIPN and 16 for CIPN; *p*-value = 0.048), indicating that the chemotherapy-naïve patients were more homogenous in terms of NGF depletion.

**Table 2:** Measured NGF levels (mean level with standard deviation in parentheses) according to the subgroups.

	No-CIPN		CIPN		*p-*value†
	Baseline NGF (pg/ml)	Follow-up NGF (pg/ml)	Baseline NGF (pg/ml)	Follow-up NGF (pg/ml)	
Previous chemotherapy					
Yes (n = 18), mean(SD)	12.2(6.61)	8.03(5.73)	9.4(5.55)	5.67(4.45)	0.964
No (n = 27), mean(SD)	8.26(3.43)	10.34(7.98)	10.60(5.83)	7.25(4.19)	0.048*
Disease					
Multiple myeloma (n = 32), mean(SD)	9.68(4.75)	9.98(8.23)	10.31(5.92)	5.54(3.84)	0.063
Lymphoma (n = 11), mean(SD)	7.22(3.35)	7.57(4.21)	10.42(4.93)	8.66(4.64)	0.614
Intervention during the study					
Bortezomib only (n = 22), mean(SD)	11.2(5.12)	7.84(7.22)	11.1(5.49)	7.15(3.34)	0.810
Thalidomide only (n = 7), median [IQR]	4.69[3.99–5.39]	10.1[9.67–10.5]	5.85[3.81–6.97]	0.80[0.69–1.56]	0.095
Vincristine only (n = 10), mean(SD)	9.62(6.09)	8.44(4.13)	12.34(4.47)	7.11(5.77)	0.300
Combination (n = 6), median [IQR]	9.21[7.82–11.1]	8.86[7.57–19.2]	4.56[3.95–11.6]	12.2[9.37–12.4]	0.700
Response					
CR (n = 10), mean(SD)	12.4(6.41)	7.17(5.79)	12.6(7.06)	5.90(5.12)	0.686
PR (n = 26), mean(SD)	9.28(4.86)	8.00(5.50)	9.39(5.46)	7.21(4.18)	0.682
Others (n = 9), median [IQR]	8.46[5.87–10.70]	12.0 [11.4–20.8]	8.47[7.08–10.40]	4.70[4.07–7.19]	0.095

*p-value of <0.05 is considered statistically significant.

† *p*-value calculated by the comparison of △NGF (follow-up NGF level–baseline NGF level) between the CIPN group and the no-CIPN group.

In the patients with multiple myeloma, the △NGF levels tended to decrease in the CIPN group (n = 12 for no-CIPN and 20 for CIPN; p-value = 0.063). This tendency was not found in the patients with lymphoma (n = 4 for no-CIPN and 7 for CIPN, *p*-value = 0.614). When we focused on the patients with newly diagnosed multiple myeloma (n = 8 for no-CIPN and 11 for CIPN), the NGF levels were significantly decreased in the CIPN group (Fig 2B; △NGF = +2.52 ± 8.39 pg/ml for No-CIPN and −4.14 ± 4.87 pg/ml for CIPN; *p*-value = 0.043).

Next, we compared the △NGF between the CIPN and no-CIPN groups according to the chemotherapeutic agents prescribed during this study. No drug was identified to make any changes to NGF levels. In addition, the △NGF levels were not associated with the patients’ clinical responses to the chemotherapy (complete response, partial response, and no response).

Lastly, we investigated the correlation between the △NGF levels in the CIPN group and the pain score (the BPI-SF subjective pain score). The △NGF levels in the patients with an average pain score of 1–3 (n = 17) and 4–5 (n = 11) were −3.67 ± 5.78 pg/ml, and −3.27 ± 5.78 pg/ml, respectively, and there was no correlation between the △NGF levels and the average pain score (Pearson’s coefficient = 0.053, *p*-value = 0.790). Similarly, the △NGF levels in the CIPN group with highest pain score of 1–3 (n = 13), 4–6 (n = 9), and 7–10 (n = 6) during the last 24 hours were −5.16 ± 6.02 pg/ml, −1.09 ± 5.84 pg/ml, and −3.58 ± 4.19 pg/ml, respectively, and there was no correlation between the △NGF levels and the highest pain score (Pearson’s coefficient = 0.280, *p*-value = 0.149).

## Discussion

This study shows that the depletion of NGF occurs during the development of CIPN in patients with hematologic malignancy. In addition, we observed that the depletion occurs more prominently in patients without previous chemotherapy, especially in patients with newly diagnosed multiple myeloma.

With the observation that the decreasing tendency of △NGF in the CIPN group was shown in multiple myeloma than other diseases, we considered that the chemotherapeutic drugs for multiple myeloma, such as bortezomib or thalidomide, could cause NGF-dependent CIPN. In the subgroup analysis for patients treated with bortezomib, △NGF levels were not significantly different between the CIPN and no-CIPN groups. However, for the patients treated with thalidomide, △NGF levels showed decreasing tendency only in the CIPN group. We could not show statistical significance in the analysis, because the number of patients who received thalidomide was only seven. Thalidomide predominantly causes axonal sensory neuropathy, but it was usually irreversible which suggests that thalidomide may damage neuronal body in dorsal root ganglia via its antiangiogenic effects [24–26]. Platinum compounds are also well-known to damage dorsal root ganglia neurons by alkylating DNAs, and the association of NGF and CIPN was documented in patients treated with CIPN [25, 27]. Given the current data and the trophic role of NGF in the peripheral nerve, we think of possibility that NGF is declined in CIPN patients treated with neuronopathic drugs. However, well-designed further studies are needed.

There were several factors other than NGF in the regulation of neuronal survival and maintenance, such as insulin-like growth factor and glia-derived neurotrophic factor[28, 29]. Considering that we could not find any difference of △NGF in the CIPN and no-CIPN groups in a few subgroup analyses including previously treated patients, lymphoma patients, and patients’ groups according to the treatment response, it would be helpful to measure and compare the changes of other neurotropic factors in further studies.

Although this study newly showed the association of NGF and CIPN in hematologic patients, there are some limitations. First, CIPN patients were diagnosed based on patients’ subjective symptoms, neurologic examinations, and laboratory blood tests, but more objective tests such as quantitative sensory test, corneal confocal microscopy, and intraepidermal nerve fiber density were not included [30, 31]. Second, 40% of study population had experience of chemotherapy. Considering that △NGF levels between the CIPN and no-CIPN groups were significantly different in chemotherapy-naïve patients, not in chemotherapy-experienced patients, we might have shown stronger statistical associations if chemotherapy-naïve patients were only included. Third, the exact mechanism of the decreased NGF after chemotherapy is not answered here and warrants further investigation.

Decrease of NGF levels was previously reported in patients with newly developed peripheral neuropathy, but exogenous NGF administration has not shown therapeutic effects for peripheral neuropathy, yet. For diabetic neuropathy, randomized, double-blind, placebo-controlled, large-scale phase 3 study of 1019 patients failed to show beneficial therapeutic effects of recombinant human nerve growth factor (rhNGF) [32]. Phase II clinical trials for rhNGF for HIV-related peripheral neuropathy showed effective at ameliorating neuropathic symptoms, but further trial did not proceed because of the failure in diabetic patients [33, 34]. For CIPN, rhNGF improved results of nerve conduction study on cisplatin neurotoxicity in rodents [14, 17]. In addition, p53 pan-neurotrophin receptor (*p*75^*NTR*^), one of NGF receptors, agonist also decreased CIPN incidence [35]. However, well-designed human trial has not been performed, probably because of uncertainty. Considering theses negative results, we thought that rhNGF could not reverse already developed CIPN. However, the negative results do not exclude the possibility of NGF as a prognostic role.

Interestingly, the role of NGF in peripheral neuropathy is not simple [36]. Patients with diabetic neuropathy showed not only decreased NGF level as described previously, but also increased level of NGF [37, 38]. Exogenous rhNGF administration also caused hyperalgesia and allodynia. It implies that excessive NGF level as well as deprivation of NGF level is associated with pain aggravation. Thus, some clinical trials using anti-NGF therapy such as tanezumab are recently in progress for alleviating sensitivity to pain [39][40].

CIPN becomes increasingly important in cancer survivors. This importance is highlighted in multiple myeloma patients, because the median survival of multiple myeloma is becoming longer than ever before with the advent of new therapeutic agents such as proteasome inhibitors and immunomodulatory agents. However, no one knows which patients will develop CIPN. When developed, approximately 20% of patients have persisting pain more than 6 months[41]. The current standard of care for CIPN is duloxetine[42], and a majority of the patients have intractable pain that disturbs daily activities. Our study showed that the NGF levels of CIPN patients were considerably decreased after chemotherapy. Also, in newly diagnosed multiple myeloma patients, the depletion of NGF was more prominent. This finding suggests the possibility of NGF as a prognostic biomarker for CIPN in multiple myeloma patients, although further research is necessary.
